# Image-Guided Pediatric Surgery Using Indocyanine Green (ICG) Fluorescence in Laparoscopic and Robotic Surgery

**DOI:** 10.3389/fped.2020.00314

**Published:** 2020-06-17

**Authors:** Ciro Esposito, Alessandro Settimi, Fulvia Del Conte, Mariapina Cerulo, Vincenzo Coppola, Alessandra Farina, Felice Crocetto, Elisabetta Ricciardi, Giovanni Esposito, Maria Escolino

**Affiliations:** ^1^Pediatric Surgery Unit, Federico II University of Naples, Naples, Italy; ^2^Urology Unit, Federico II University of Naples, Naples, Italy; ^3^Pharmacy Unit, Federico II University of Naples, Naples, Italy

**Keywords:** indocyanine green, fluorescence, technology, children, laparoscopy, robotics

## Abstract

**Background:** Indocyanine green (ICG)-guided near-infrared fluorescence (NIRF) has been recently adopted in pediatric minimally invasive surgery (MIS). This study aimed to report our experience with ICG-guided NIRF in pediatric laparoscopy and robotics and evaluate its usefulness and technique of application in different pediatric pathologies.

**Methods:** ICG technology was adopted in 76 laparoscopic and/or robotic procedures accomplished in a single division of pediatric surgery over a 24-month period (January 2018–2020): 40 (37 laparoscopic, three robotic) left varicocelectomies with intra-operative lymphography; 13 (10 laparoscopic, three robotic) renal procedures: seven partial nephrectomies, three nephrectomies, and three renal cyst deroofings; 12 laparoscopic cholecystectomies; five robotic tumor excisions; three laparoscopic abdominal lymphoma excisions; three thoracoscopic procedures: two lobectomies and one lymph node biopsy for suspected lymphoma. The ICG solution was administered into a peripheral vein in all indications except for varicocele and lymphoma in which it was, respectively, injected into the testis body or the target organ. Regarding the timing of the administration, the ICG solution was administered intra-operatively in all indications except for cholecystectomy in which the ICG injection was performed 15–18 h before surgery.

**Results:** No conversions to open or laparoscopy occurred. No adverse and allergic reactions to ICG or other postoperative complications were reported.

**Conclusions:** Based upon our 2 year experience, we believe that ICG-guided NIRF is a very useful tool in pediatric MIS to perform a true imaged-guided surgery, allowing an easier identification of anatomic structures and an easier surgical performance in difficult cases. The most common applications in pediatric surgery include varicocele repair, difficult cholecystectomy, partial nephrectomy, lymphoma, and tumors excision but further indications will be soon discovered. ICG-enhanced fluorescence was technically easy to apply and safe for the patient reporting no adverse reactions to the product. The main limitation is represented by the specific equipment needed to apply ICG-guided NIRF in laparoscopic procedures, that is not available in all centers whereas the ICG system Firefly® is already integrated into the robotic platform.

## Introduction

Near-infrared fluorescence (NIRF) is a common application of fluorescence image-guided surgery (FIGS) (1–3). Use of NIRF requires the injection of a specific dye, indocyanine green (ICG), that first received Food and Drug Administration (FDA) approval to study hepatic and cardiac function in humans, and therefore it was adopted off-label for lymphatic targeting (4–6). Recently, ICG-enhanced fluorescence was proved to be very useful to improve intra-operative anatomic view during laparoscopy or robotics and thus increase patients' safety during difficult surgical procedures (7–9). Regarding the mechanism of action, fluorescence is produced when the water-soluble dye, ICG, is excited using a light of a specific wavelength in the NIR spectrum (~820 nm) and is visualized using specific optics and camera systems (7, 10). The last generation of robot, such as da Vinci Xi (Intuitive Surgical, Sunnyvale, CA), is equipped with a software for NIRF detection, named Firefly® (Novadaq Technologies, Mississauga, ON), and its utilization is directly controlled by the console surgeon (11). ICG is almost entirely metabolized by the liver and excreted into the bile, so the biliary indications are the most obvious (12, 13). Other common applications of ICG fluorescence in adults included sentinel lymph node mapping, angiographic study, and perfusion assessment of various organs or tumors in abdominal, thoracic, or urological surgery (14–17).

The use of ICG fluorescence imaging in pediatric patients is just at embryonal stage. In pediatric patients, use of ICG lymphography has been described for treatment of varicocele and neonatal chylothorax or other lymphatic malformations (18–20). Different useful applications of this technology have been identified in children such as visualization of biliary tract anatomy and assessment of vascular territory, tissue perfusion, and tumor localization in various organs. However, analyzing the international literature, there is still limited evidence about the usefulness of ICG-guided NIRF imaging in such indications (21–23). Furthermore, as most knowledge and data were generated from adult experience, the dosage, and timing of injection were difficult to standardize in children.

This study aimed to report the results of our experience with ICG-enhanced fluorescence imaging in pediatric minimally invasive surgery (MIS)—laparoscopy and thoracoscopy—and in robotics. ItIt also aimed to define the indications and to standardize dosage, timing, and modality of application of ICG-guided NIRF in different pediatric pathologies.

## Materials and Methods

ICG technology was adopted in 76 laparoscopic and/or robotic procedures accomplished in a single division of pediatric surgery over a 24-month period (January 2018–2020): 40 (37 laparoscopic, three robotic) left varicocelectomies with intra-operative lymphography; 13 (10 laparoscopic, three robotic) renal procedures: seven partial nephrectomies, three nephrectomies, and three renal cyst deroofings; 12 laparoscopic cholecystectomies; five robotic tumor excisions; three laparoscopic abdominal lymphoma excisions; three thoracoscopic procedures: two lobectomies and one lymph node biopsy for suspected lymphoma.

Regarding the equipment, a specific camera system and a specific 0- or 30-degree laparoscope equipped with a special filter for detection of both NIR light and standard white light (KARL STORZ SE & Co. KG, Tuttlingen, Germany) were used in all laparoscopic and thoracoscopic procedures (21). There is a specific software that reproduces the NIR image in different colors through selection of different view modes: green or blue (CLARA + CHROMA mode) or white (SPECTRA A mode). The specific view mode can be selected by the surgeon through the buttons on the camera head during the initial setting. Switching from white light mode to NIRF is directly activated by the surgeon through foot-pedal pushing. The visualization of both white and NIR light is enhanced by a professional system of image visualization (IMAGE1 S system, KARL STORZ SE & Co. KG, Tuttlingen, Germany). In robotics, the console surgeon can switch from standard white light to NIRF by pressing a button on the robotic joystick. Finally, the ICG dye (Verdye, Pulsion Medical Systems, Munich, Germany), available in vials (25 mg/ml), was adopted in all the procedures. As indicated by the FDA recommendations, the routine dose for ICG should be 2.5 mg and 1.25 mg in children and infants, respectively (11). For all indications, ICG vial was diluted with sterile water to create a 2.5 mg/ml solution. The ICG solution was administered into a peripheral vein in all indications except for varicocele and lymphoma in which it was, respectively, injected into the testis body or the target organ. Regarding the timing of the administration, the ICG solution was administered intra-operatively in all indications except for cholecystectomy in which the ICG injection was performed 15–18 h before surgery.

The study was reviewed and approved by ethics committee of Federico II University of Naples, in Naples, Italy (216/2018). The patients' legal guardians provided written informed consent to participate in this study.

Details of dosage, timing, and modality of administration of ICG are reported for each surgical procedure (Table 1).

**Table 1:** Technical details of ICG applications in pediatric surgical procedures.

**Indication**	**Dosage**	**Timing of administration**	**Modality of administration**	Advantage*
Varicocele repair	3.125 mg/ml	Intra-operatively	Intra-parenchymal testicular injection	++++
Nephrectomy	0.3 mg/ml/kg	Intra-operatively	Intravenous injection	++
Partial nephrectomy	0.3 mg/ml/kg	Intra-operatively	Intravenous injection	++++
Renal cyst deroofing	0.3 mg/ml/kg	Intra-operatively	Intravenous injection	+++
Cholecystectomy	0.4 mg/ml/kg	16–18 h pre-operatively	Intravenous injection	++++
Lymphoma/abdominal tumors removal	0.5 mg/ml/kg	Intra-operatively	Intravenous injection	++++
Ovarian tumor removal	0.5 mg/ml/kg	Intra-operatively	Intravenous injection	+++
Lobectomy	0.25 mg/ml/kg	Intra-operatively	Intravenous injection	+
Thoracic lymph node biopsy	0.5 mg/ml/kg	Intra-operatively	Intra-parenchymal lung injection	+++

* *Advantage scoring: +none; ++low; +++average; ++++high*.

### Urology

#### Varicocele Repair

**Figure 1:**
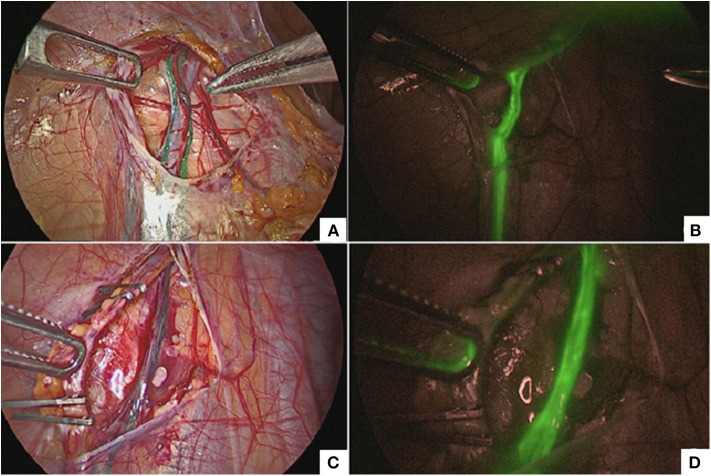
Lymphatics sparing at standard white light **(A)** and ICG-guided NIRF **(B)**. Clipping and division of the spermatic bundle at standard white light **(C)** and ICG-guided NIRF **(D)**.

After pneumoperitoneum induction, a bottle of ICG with a concentration of 25 mg was reconstituted with 8 ml of sterile water, and 2 ml (6.25 mg) of this solution were directly injected into the body of the left testicle using a 23G needle. Under NIR light, the lymphatics appeared fluorescent (blue or green with CLARA + CHROMA mode, white with SPECTRA A mode and green with FIREFLY®) and were easily isolated and spared (Figure 1). Thereafter, both spermatic vein and artery were ligated using 5-mm titanium clips and sectioned following the Palomo's principle (Figure 1). Furthermore, the lymphatics were also clearly identifiable under standard white light as they appeared green or blue colored.

All operative steps are reported in Video 1.

#### Nephrectomy/Partial Nephrectomy

**Figure 2:**
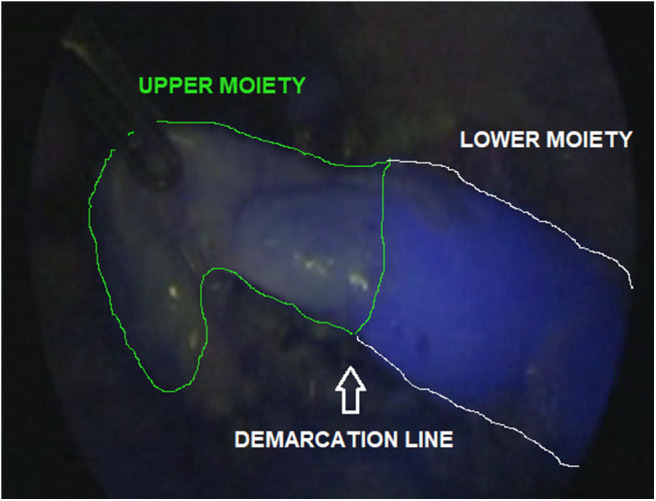
ICG-guided NIRF aided to identify the dissection plane between upper and lower moiety during upper pole partial nephrectomy.

The ICG solution (dosage 0.3 mg/ml/kg) was injected intravenously, just after the division of the Gerota's fascia and ICG-guided NIRF allowed to visualize the vascularization of the kidney within 5 min. During nephrectomy, ICG-guided NIRF was adopted to visualize the vascular anatomy before hilar vessel control, especially in patients who had dense adhesions of the perirenal tissues. In case of partial nephrectomy, ICG-enhanced fluorescence was very useful to identify the main hilar vessels and the vessels supplying the upper/lower moiety. After division of vessels supplying the upper/lower moiety, ICG-guided NIRF aided to define the dissection plane between the two moieties (Figure 2) and finally check the vascularization of the normal moiety following the resection of the non-functioning pole.

#### Renal Cyst Deroofing

The ICG solution (dosage 0.3 mg/ml/kg) was injected intravenously, just after the division of the Gerota's fascia. In a matter of about 60 s following the injection, ICG-enhanced fluorescence allowed to clearly distinguish the non-vascularized cyst dome from the vascularized renal parenchyma. Once identified and exposed, the cyst was punctured with a needle, that was introduced transabdominally and the liquid content was aspirated. The cyst's roof was resected and the cyst's concavity was finally wadded using a pedicled flap of perirenal fat tissue.

### Hepatobiliary Surgery

#### Cholecystectomy

**Figure 3:**
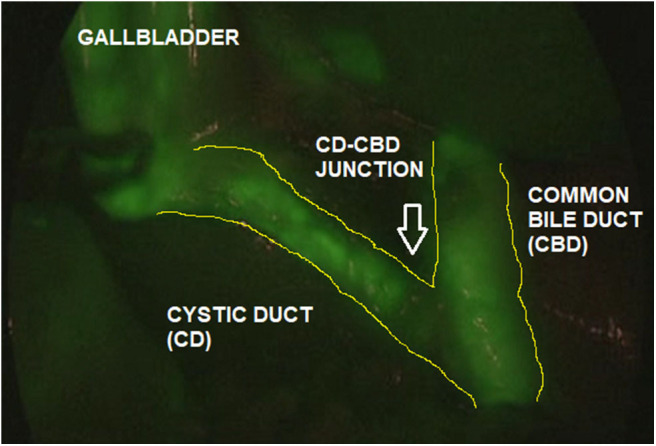
ICG-guided NIRF aided to identify the biliary anatomy (Cystic Duct, CD; Common Bile Duct, CBD; CD-CBD junction) during laparoscopic cholecystectomy.

**Figure 4:**
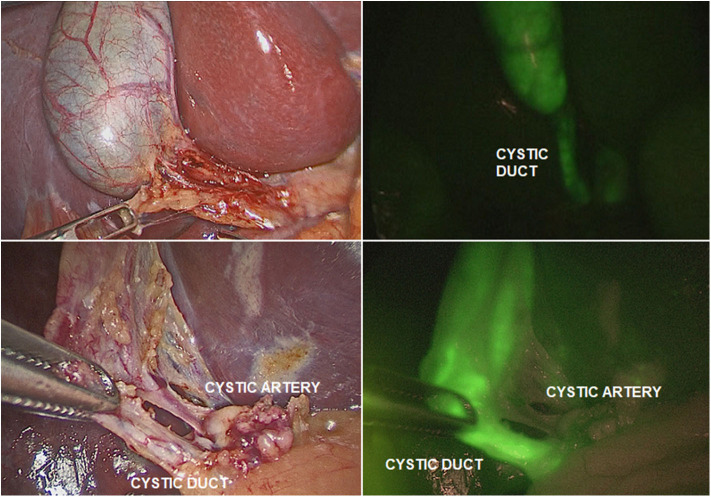
ICG-guided NIRF was helpful to identify the different structures (cystic duct; cystic artery) in presence of adhesions or inflammation.

The ICG solution (dosage 0.4 mg/ml/kg) was administered intravenously 15–18 h pre-operatively. This interval time between ICG administration and surgery assured that most of the dye was exclusively concentrated into the extrahepatic biliary structures and absent in the liver parenchyma. ICG-guided NIRF aided to clearly visualize the gallbladder and the biliary structures including the cystic duct (CD), the common bile duct (CBD), and especially the CD-CBD junction, despite the presence of plenty adipose tissue, inflammation and dense tissue adhesions (Figure 3). ICG technology was very helpful to achieve the Critical View of Safety (CVS) and avoid the risk of iatrogenic biliary or vascular injuries. The CVS was defined by three criteria: (1) the hepatocystic triangle was freed of adipose and fibrotic tissue; (2) the lower portion of the gallbladder was separated from the liver in order to expose the cystic plate; and (3) only the CD and the cystic artery entered the gallbladder (Figure 4) (24). ICG-enhanced fluorescence was also useful to identify the thin dissection plane between the gallbladder and the liver during its final detachment from the liver bed.

### Oncology

#### Lymphoma and Abdominal Tumors Removal

For this indication, the ICG solution (dosage 0.5 mg/ml/kg) was intra-operatively injected into a peripheral vein and allowed a “real-time” assessment of bowel perfusion. Additionally, ICG-guided NIRF aided to visualize the vascularization of the neoplastic mass, to define the optimal level of resection in case of mesenteric division, and to detect the nodes to biopsy or to resect.

#### Ovarian Tumors Removal

**Figure 5:**
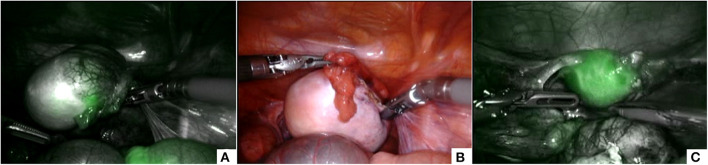
ICG-guided NIRF was useful to identify the tumor mass that appeared hypo-fluorescent **(A)**, to respect the resection edges during removal **(B)**, and to check the vascularization of the uterus following the resection **(C)**.

Intra-operatively, the ICG solution (dosage 0.5 mg/ml/kg) was injected intravenously and in a matter of about 20–30 s following the injection, ICG-enhanced fluorescence allowed to clearly identify the tumor mass that appeared hypo-fluorescent compared with the normal salpinx and ovarian parenchyma (Figure 5A). ICG-guided NIRF aided the surgeon to identify and respect the resection edges during the removal of the tumor mass (Figure 5B) and to check the vascularization of the salpinx and the uterus following the mass resection (Figure 5C).

### Thoracic Surgery

#### Thoracoscopic Lobectomy

Intra-operatively, the ICG solution (dosage 0.25 mg/ml/kg) was injected into a peripheral vein, with the aim to identify the inter-segmental plane between the cystic malformation and the normal lung parenchyma and better define the resection margins.

#### Lymph Node Thoracoscopic Biopsy

**Figure 6:**
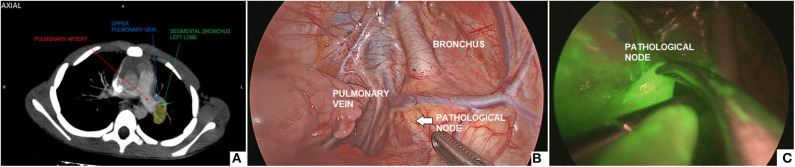
Thoracoscopic biopsy of a 2-cm hilar lymph node: pre-operative CT imaging **(A)**, intra-operative view **(B)**, ICG-guided NIRF imaging **(C)**.

Intra-operatively, the ICG solution (dosage 0.5 mg/ml/kg) was injected into the lung parenchyma with a metallic needle, that was introduced through a 5-mm trocar. ICG-guided NIRF was helpful to localize the pathological node to biopsy (Figure 6).

## Results

All the surgical procedures were performed under general anesthesia and were completed laparoscopically, thoracoscopically, or robotically without conversions. No patients had a previous history of allergy to iodide. No adverse and allergic reactions to ICG were reported.

Other results are reported separately for each surgical procedure.

### Urology

#### Varicocele Repair

Left varicocelectomy with intra-operative lymphography was accomplished via laparoscopic (*n* = 37) and robot-assisted (*n* = 3) approach. The mean patient age at surgery was 16.8 years (range 8–18). The indications for surgery were high grade varicocele associated with left testicular hypotrophy in all cases and with symptoms (testicular pain and/or discomfort) in 26/40 patients (65%). The average length of surgery was 16 ± 9 min. At the longest follow-up of 24 months, no persistence and/or recurrence of disease or testicular atrophy or hydrocele were observed. Furthermore, no adverse reactions of the left testicle such as postoperative edema, hematoma, orchitis, and/or pain, were reported.

#### Nephrectomy/Partial Nephrectomy

ICG-enhanced fluorescence was adopted during laparoscopic partial nephrectomy (*n* = 7) and laparoscopic nephrectomy (*n* = 3). There were four girls and six boys and the mean patient age at surgery was 5.7 years (range 1–17). The indications for nephrectomy included non-functioning hydronephrotic kidney (*n* = 2) and non-functioning kidney due to vesico-ureteral reflux (VUR) nephropathy (*n* = 1). The indications for partial nephrectomy included non-functioning symptomatic obstructive upper pole moiety (*n* = 4) and non-functioning symptomatic lower pole moiety associated with VUR (*n* = 3). The average operative time was 78.5 ± 8 min. No intra- and post-operative complications occurred in all patients.

#### Renal Cyst Deroofing

Robotic deroofing of simple renal cyst (SRC) was performed in three boys. The mean patient age at surgery was 10.3 years (range 6–15). The median pre-operative cyst size was 70 mm (range 42–90) and all cysts were identified as II grade according to Bosniak classification. All patients were symptomatic with recurrent flank pain. The average operative time was 75 ± 11 min including surgical and docking time. No intra- and post-operative complications occurred in all patients, who remained asymptomatic throughout the follow-up period.

### Hepatobiliary Surgery

#### Cholecystectomy

An elective laparoscopic 4-trocars cholecystectomy was accomplished in twelve obese adolescents (four boys and eight girls) with a mean age at surgery of 16.8 years (range 12–17) and a mean BMI of 33.1 ± 2.0 kg/m^2^. The average length of surgery was 62 ± 15 min. No complications occurred intra- or post-operatively. In one patient affected by Crigler–Najjar syndrome type II, who was under phenobarbital treatment, we noticed intra-operatively a weak fluorescence of the extrahepatic biliary structures due to the increased hepatic metabolism of ICG induced by phenobarbital. In this patient, an additional administration of ICG (dosage 0.4 mg/ml/kg) was needed intra-operatively in order to visualize the cystic artery.

### Oncology

#### Lymphoma and Abdominal Tumors Removal

ICG-guided NIRF was adopted intra-operatively in three patients (one girl and two boys), who underwent laparoscopic excision of abdominal lymphoma. The mean patient age at surgery was 4.1 years (range 2–8). The average duration of surgery was 138 ± 8 min. No complications occurred intra- or post-operatively.

#### Ovarian Tumors Removal

ICG-guided NIRF was adopted during robot-assisted removal of five ovarian tumors. The mean patient age at surgery was 13.5 years (range 11–16). The mean pre-operative size of adnexal mass was 8.5 cm (range 7–15). The average length of surgery was 78 ± 12 min. No complications occurred intra- or post-operatively. The histological diagnosis included mature teratoma (*n* = 3) and seromucinous cystadenoma (*n* = 2).

### Thoracic Surgery

#### Thoracoscopic Lobectomy

ICG-enhanced fluorescence was adopted during thoracoscopic left lower lobectomy in two patients (one girl and one boy), who were affected by congenital cystic adenomatoid malformation (*n* = 1) and pulmonary extra-lobar sequestration (*n* = 1). The mean patient age at surgery was 15.5 months (range 11–20). We found no true benefits of ICG-guided NIRF during these procedures, because we were not able to identify a clear demarcation line between the cystic malformation and the normal parenchyma.

#### Lymph Node Thoracoscopic Biopsy

ICG-enhanced fluorescence was also adopted during thoracoscopic biopsy of a 2-cm hilar lymph node with suspicion of lymphoma in a 6 years-old boy. The length of surgery was 68 min. No complications occurred intra- or post-operatively. Histology excluded the presence of malignant cells and the patient is currently being followed-up by pediatric oncologists.

## Discussion

The use of intra-operative NIRF imaging using ICG has been recently described as a very useful tool in decision-making strategy during challenging surgical procedures with a growing evidence in the adult literature (7–9). The applications of ICG-enhanced fluorescence in adults are wide and include oncologic and non-oncologic indications in different specialties such as colorectal, vascular, hepatobiliary, urological, and thoracic surgery with very promising results (11–16). ICG-enhanced fluorescence has also been employed in pediatric MIS with the aim to provide a more precise visualization of intra-operative anatomy (21). However, there is still very limited evidence in the pediatric literature and the indications of ICG-guided NIRF are not yet so clear in pediatric patients (21–24). Based upon our preliminary experience with application of ICG-enhanced fluorescence in pediatric surgery and pediatric urology, we would make some general considerations about its use in children.

As ICG is entirely excreted into the bile, the identification of the anatomic structures of the Calot's triangle during hepatobiliary surgery represented one of the most frequent and worthwhile applications of ICG-guided NIRF in both adults and children (13, 24, 25). In fact, iatrogenic biliary damages, commonly due to the scarce intra-operative visualization of biliary structures, probably represent the most feared complications of cholecystectomy (26). Inexperience, inflammation, and aberrant anatomy are key risk factors (27). It has been reported that the intra-operative real-time virtual cholangiography, provided by ICG-guided NIRF, allowed to perform a precise and safe dissection of the Calot's triangle and decreased the risk of iatrogenic biliary duct injury (13, 14). In our experience, ICG-guided NIRF aided to clearly visualize the gallbladder and the biliary structures including the CD, the CBD, and especially the CD-CBD junction in all cases, despite the presence of plenty adipose tissue, inflammation, and dense tissue adhesions (Figures 3, 4). ICG technology was very helpful to achieve the CVS and further decrease the incidence of iatrogenic biliary or vascular injuries. We recently published our 25-year experience with laparoscopic cholecystectomy using both standard laparoscopic technique and ICG-guided NIRF (24). We reported a postoperative complications rate of 1.9%: one bleeding from the cystic artery, one dislocation of the clips on the CD, and two iatrogenic injuries to the main bile duct (24). All the complications occurred in patients who were operated using standard laparoscopic technique. No postoperative complications occurred in patients who were operated using intra-operative ICG-guided fluorescence imaging (24).

The optimal timing of ICG administration in such indication has also been long debated (13); in our series, the ICG solution (dosage 0.4 mg/ml/kg) was administered intravenously 15–18 h pre-operatively, allowing a successful visualization of the biliary anatomy in the totality of patients. This interval time between ICG injection and surgery assured that most of the dye was exclusively concentrated into the extrahepatic biliary structures, so as to avoid the background hyper-fluorescence of the liver that was typically observed when the injection was performed just before the procedure. We also observed a poor visualization of the extra-hepatic biliary structures under NIRF in one patient affected by Crigler–Najjar syndrome type II who was under treatment with phenobarbital. The weak fluorescence of the biliary tree in this patient was due to the pharmacodynamics of the phenobarbital, that accelerated the liver metabolism and the biliary excretion of the dye. Based upon this finding, we suggest that the timing of ICG injection should be corrected in patients under phenobarbital treatment and should be performed at least 7–9 h prior to the surgery instead of 15–18 h pre-operatively, as routinely done.

Another very good indication of ICG-enhanced fluorescence in pediatric surgery is intra-operative lymphography during laparoscopic Palomo varicocelectomy (17). In our experience, ICG-enhanced fluorescence allowed to identify lymphatics in 100% of cases (Figure 1) and to perform a lymphatic sparing procedure avoiding the risk of postoperative hydrocele. Before the advent of ICG-enhanced fluorescence, we already performed lymphatic-sparing varicocele repair using intradartoic/ intratesticular injection of isosulfan blue dye. As we already published, we recorded no postoperative hydrocele also using this last technique (28). Thereafter, we decided to adopt ICG to perform lymphography and we standardized the injection technique (18). We found two main advantages of using ICG over isosulfan blue: first, ICG, being metabolized by the liver, did not modify the urines' color, and secondly ICG did not cause any color changes of the skin in the injection site (17). Currently, ICG-enhanced fluorescence has become our first choice to perform lymphatic-sparing Palomo procedure.

ICG technology may be useful during partial nephrectomy (29). In this indication, ICG-guided NIRF aided to exactly identify the vascularization of both normal and non-functioning moieties (Figure 2). After division of the vessels supplying the upper/lower moiety, ICG-guided NIRF aided to define the dissection plane between the two moieties, thus decreasing the risk of injury to the normal moiety or post-operative urinary leakage. Furthermore, it was also useful to check the vascularization of the normal moiety following the resection of the affected moiety.

Another interesting clinical application of ICG-enhanced fluorescence was for oncological indications (30, 31). In our series, ICG-guided NIRF was very helpful to detect also smaller tumors and to visualize the vascular anatomy and the resection plane of the tumor so as to perform a safe and precise dissection and removal of the mass.

Regarding nephrectomy and lobectomy, our preliminary experience using ICG-guided NIRF in such procedures demonstrated no clear advantages, and probably a larger case series is needed to achieve further evidence about usefulness of ICG technology for such indications. Considering the paucity of the available data that made dosage and timing of administration difficult to standardize in children (23), we set up modality, timing, and dosage of administration of ICG for each indication. Regarding the modality of administration, the ICG dye was injected intravenously in most indications; in few cases such as lymphatic sparing varicocele repair or oncologic procedures, the dye must be directly injected in the target tissue or organ. Regarding the timing of administration, the product was injected during surgery in most indications except for cholecystectomy, in which the ICG injection was performed at least 16–18 h prior to the surgery. Regarding the dosage of ICG, we standardized the dosage for each procedure (Table 1); in our clinical practice, the dosages ranged between 0.3 and 0.5 mg/ml/kg and were much lower than the reported toxicity levels (3, 7).

Based upon our preliminary experience, we believe that ICG-enhanced fluorescence provided several advantages in pediatric patients allowing to better identify the surgical anatomy and fasten the surgical procedure while maintaining the patient's safety. This technique was no time-consuming since it just required an intravenous or intra-parenchymal injection of ICG solution and fluorescence of target tissue/organ was visualized in real-time intra-operatively (7). Furthermore, ICG use was clinically safe since no allergy and other adverse systemic reactions or any testicular injury related to the intra-testicular ICG injection were reported in the early or late postoperative course (17, 21). Use of ICG-guided NIRF was also cost-effective; in fact, since the operating room was already equipped with the specific camera system and laparoscopic equipment for NIR light detection and the robotic platform was already integrated with the Firefly® software for NIR light detection, use of NIRF in both laparoscopy and robotics did not require any adjunctive costs except for the ICG vial (cost about 40 eur).

However, ICG technology does have its limitations: first, the special equipment required for its use in laparoscopic surgery may not be available in all centers. This problem is overcome in robotic surgery, since the ICG software Firefly® is already integrated into the robotic platform (11). The sensitivity and visualization of ICG are significantly affected by the depth and size of the lesion (23). In fact, very small lesions are difficult to detect if the intensity of fluorescence is too weak (20, 23). Finally, the use of ICG is not recommended in patients who reported allergy to iodides since ICG contains sodium iodide (23).

Based upon our 2 year experience, we believe that ICG-guided NIRF is a very useful tool in pediatric MIS to perform a true imaged-guided surgery, allowing an easier identification of anatomic structures and an easier surgical performance in difficult cases. The most common applications in pediatric surgery include varicocele repair, difficult cholecystectomy, partial nephrectomy, lymphoma, and tumors excision but further indications will be soon discovered. ICG-enhanced fluorescence was technically easy to apply and safe for the patient reporting no adverse reactions to the product. The main limitation is represented by the specific equipment needed to apply ICG-guided NIRF in laparoscopic procedures, that is not available in all centers whereas the ICG system Firefly® is already integrated into the robotic platform.

## Data Availability Statement

All datasets presented in this study are included in the article/Supplementary Material.

## Ethics Statement

The study was reviewed and approved by ethics committee of Federico II University of Naples, in Naples, Italy. The patients' legal guardians provided written informed consent to participate in this study.

## Author Contributions

CE contributed conception and design of the study and wrote the first draft of the manuscript. AS contributed conception and design of the study and wrote sections of the manuscript. FDC, MC, VC, AF, FC, ER, GE, and ME organized the database and wrote sections of the manuscript. All authors contributed to manuscript revision, read, and approved the submitted version.

## Conflict of Interest

The authors declare that the research was conducted in the absence of any commercial or financial relationships that could be construed as a potential conflict of interest.
